# Supplementary material for: Survival Is Related to Estrogen Signal Transduction Pathway Activity in Postmenopausal Women Diagnosed with High-Grade Serous Ovarian Carcinoma
Source: Cancers (Basel). 2021 Oct 12;13(20):5101. doi: 10.3390/cancers13205101 (PMC8533979; doi:10.3390/cancers13205101)
Supplement: Supplementary file 1 [file cancers-13-05101-s001.zip › cancers-1380917-supplementary.pdf]

Supplementary tables

Table S1. Clinicopathological characteristics of the subgroups based on ER pathway activity in postmenopausal HGSC patients.

Variable	Quartile 1<br>*n*=17 (%)	Quartile 2<br>*n*=17 (%)	Quartile 3<br>*n*=17 (%)	Quartile 4<br>*n*=16 (%)	P-value*
**Age at diagnosis**					0.828
Median (IQR)	65 (63 – 72)	65 (61 – 71)	67 (59 – 73)	64 (58 – 72)	
**FIGO stage**					1.000
IIIC	15 (88)	15 (88)	15 (88)	14 (87)	
IV	2 (12)	2 (12)	2 (12)	8 (13)	
**CA125 concentration at diagnosis**					0.220
Median (IQR)	340 (68–737)	690 (242–2057)	245 (59–603)	288 (163–1032)	
Missing (n)	-	1 (6)	1 (6)	-	
**CA125 concentration after treatment**					0.826
Median (IQR)	13 (9 – 16)	12 (8 – 16)	11 (7 – 13)	12 (6 – 14)	
Missing (n)	4 (24)	3 (18)	4 (24)	2 (13)	
**Debulking type**					0.990
Primary	9 (53)	10 (59)	9 (53)	8 (50)	
Interval	7 (41)	6 (35)	8 (47)	7 (44)	
Other**	1 (6)	1 (6)		1 (6)	
**Debulking outcome**					0.910
Complete	13 (76)	11 (65)	13 (76)	11 (69)	
Optimal	3 (18)	4 (23)	2 (12)	2 (12)	
Incomplete	1 (6)	2 (12)	2 (12)	3 (19)	

* Differences in continuous variables were tested with a Mann-Whitney U test (skewed distribution). For categorical variables, P-values were obtained from a Fisher's exact test.

** Incomplete primary debulking followed by neo-adjuvant chemotherapy and afterwards a secondary interval debulking with adjuvant chemotherapy.

Abbreviations: IQR, interquartile range.
